# Clinical Significance of Haplo-Fever and Cytokine Profiling After Graft Infusion in Allogeneic Stem Cell Transplantation From Haplo-Identical Donors

**DOI:** 10.3389/fmed.2022.820591

**Published:** 2022-04-07

**Authors:** Lining Wang, Bo Dai, Wenhui Gao, Jing Wang, Ming Wan, Runshu Wang, Ling Wang, Jieling Jiang, Didier Blaise, Jiong Hu

**Affiliations:** ^1^Shanghai Institute of Hematology, Blood and Marrow Transplantation Center, Collaborative Innovation Center of Hematology, Department of Hematology, Ruijin Hospital, Shanghai Jiao Tong University School of Medicine, Shanghai, China; ^2^Shanghai Clinical Research Center, Fenglin International Centre, Shanghai, China; ^3^Department of Biostatistics, University of Michigan School of Public Health, Ann Arbor, MI, United States; ^4^Transplantation and Cell Therapy Program, Leukemia Program, Centre de Recherche en Cancérologie de Marseille, Department of Hematology, Institut Paoli-Calmettes, Aix-Marseille University, Marseille, France

**Keywords:** haplo-HSCT, CRS, cGvHD, allo-reactivation, haplo-fever

## Abstract

Allogeneic stem cell transplantation from haplo-identical donors (haplo-HSCT) has become a well-established therapeutic option for hematological malignancies. The fever of unknown origin (haplo-fever) early after the infusion of T cell repleted graft, which returned to normal right after post-transplantation cyclophosphamide (PTCy), is a unique clinical feature in patients undergoing haplo-HSCT. In the current study, the characteristics of haplo-fever and cytokine profiles during haplo-fever were retrospectively analyzed in a cohort of 37 patients undergoing T cell repleted haplo-HSCT with PTCy as graft versus host disease (GvHD) prophylaxis. In total, 33 patients (89.2%) developed haplo-fever from day 0 to day +7. Patients with high peak temperatures tended to have a lower incidence of chronic GvHD (cGvHD) (*p* = 0.07), moderate to severe cGvHD (*p* = 0.08), and superior GvHD and relapse-free survival (GRFS, *p* = 0.04). During the haplo-fever, there were significant increases in multiple cytokines, such as interferon gamma, interleukin (IL) 6, IL2, IL2 receptor, IL8, IL10, IL17, and tumor necrosis factor (TNF). The increases in IL2 receptor (*p* = 0.037) and TNF (*p* < 0.001) on day +4 were correlated with the lower risk of cGvHD. Increased TNF > 1.8055-fold on day +4 was the best predictive threshold for cGvHD, and was correlated with a lower incidence of cGvHD (*p* < 0.001), moderate to severe cGvHD (*p* = 0.003), and superior GRFS (*p* < 0.001). These observations may reflect the early reactivation of donor T cells after haplo graft infusion, which would potentially be eliminated by PTCy. Further studies with larger independent cohorts of patients are warranted, to clarify the clinical significance of haplo-fever, and day +4 TNF as a potential biomarker to predict GvHD and GRFS.

## Introduction

Allogeneic stem cell transplantation (allo-HSCT) is a potentially curative treatment for various hematological malignancies. Allo-HSCT from haplo-identical donors (haplo-HSCT) is well established as an alternative option in patients without HLA-matched related or unrelated donors (1–4). Graft versus host disease (GvHD) prophylaxis by post-transplantation cyclophosphamide (PTCy) is the mainstay in haplo-HSCT (5, 6).

Early fever development after graft infusion, followed by immediate recovery after PTCy treatment, has been reported in up to 90% of patients and is reportedly a unique clinical feature of haplo-HSCT, but only a few cases have been described in HLA-matched transplantation using PTCy for GvHD prophylaxis (7–9). In addition, symptoms resembling cytokine release syndrome (CRS) have been reported shortly after graft infusion from haplo-identical donors (8, 9). CRS-like symptoms (CLSs) described in haplo-HSCT include fever, hypoxia, hypotension, renal function impairment, and capillary leak syndrome from day 0 to day +14 post-transplantation. Generally, the CLSs were mild, but severe CLSs may be associated with delayed engraftment, a higher risk of non-relapse mortality (NRM), and inferior overall survival (10).

Early fever and CLSs may be the manifestations of the activation of allo-reactive donor T cells soon after graft infusion. Interleukin (IL) 6 is one of the key cytokines involved in CRS after chimeric antigen receptor (CAR) T cell treatment, and the use of the monoclonal anti-IL6 antibody tocilizumab was effective in treating such CRS (11, 12). With respect to CLSs in haplo-HSCT, several studies have demonstrated that IL6 was increased and the CLSs could be treated with methylprednisolone or tocilizumab (9, 10, 13). In phase I/II study, cyclosporine and mycophenolate mofetil given from day -1 instead of from day +5 in the PTCy model successfully diminished the incidence of early fever after graft infusion, further supporting an immune-based mechanism of fever and/or CLSs (14).

To clarify the symptoms and underlying immune mechanisms of early fever after haplo-HSCT (haplo-fever), the current study investigated the characteristics of febrile events and cytokine profiles from day 0 to day +7 post-transplantation in haplo-HSCT with PTCy, and analyzed the clinical effects of fever and cytokine profiles on transplant outcomes.

## Materials and Methods

### Patients

In this study, 37 consecutive adult patients enrolled in three prospective clinical trials for lymphoid malignancies, acute myeloid leukemia (AML), or myelodysplasia syndrome (MDS), and refractory AML (NCTs 04118075, 04269811, and 03882203, respectively) from June 2019 to December 2020 were included. All three aforementioned trials were approved by the Human Ethics Committee of Ruijin Hospital, and were conducted in accordance with the Declaration of Helsinki. Informed consent was obtained from all patients. All patients received unmanipulated peripheral blood stem cells (PBSCs) from haplo-identical donors. The conditioning regimens used were in accordance with the trial protocols. All patients received the same GvHD prophylaxis regimen, which included 50 mg/kg cyclophosphamide on day +3 and day +4, followed by 0.05 mg/kg/day tacrolimus from day +5 and 2.5 mg/kg anti-thymoglobulin on day +15 or day +22 after neutrophil engraftment. All patients received levofloxacin as antibiotic prophylaxis, and imipenem was given as an empirical antibiotic for febrile neutropenia. No immunosuppressive agents, such as steroid or anti-IL6, were given in addition to the routine GvHD prophylaxis during febrile events between day 0 and day +7.

### Data Collection

Data derived from daily vital signs, physical examinations, and biochemical tests were collected from databases and then, were retrospectively analyzed. Fever was defined as a temperature between 38.0 and 38.2°C on two consecutive measurements of at least 1 h apart, or any single temperature measurement of ≥38.3°C. Biochemical tests included transaminase, bilirubin, serum creatinine, serum urea, uric acid, electrolytes, and myocardial proteins. The quantitative detection of cytokines was performed from day 0 to day +7 in accordance with the trial protocol. The panel of cytokines consisted of IL1β, IL2 receptor (IL2R), IL6, IL8, IL10, tumor necrosis factor (TNF), interferon (IFN) α, IFNγ, IL5, IL2, IL17, and IL12p70. Cytokine data collected from databases were retrospectively analyzed. CRS severity was graded in accordance with the ASTCT criteria (15).

Transplantation outcome data, such as the date of engraftment, occurrence and grading of acute GvHD (aGvHD) or chronic GvHD (cGvHD), relapse of disease, graft failure, non-relapse mortality (NRM), and death, were all retrieved from the relevant databases. For original data, please contact hj10709@rjh.com.cn.

### Statistics

Between-group patient characteristics were evaluated using Fisher’s exact test for categorical variables, and *t*-tests for continuous variables. Differences in times to events between groups were estimated with Cox proportional hazards models. The cumulative incidence of cGvHD was calculated as the time from transplant to the diagnosis of cGvHD (event), relapse (competing event), primary graft failure (competing event), death not due to cGvHD (competing event), or last follow-up if still alive (censored). GvHD and relapse-free survival (GRFS) were calculated from the day of transplant to the diagnosis of grade III-IV aGvHD, moderate to severe cGvHD, mild cGvHD requiring systemic immunosuppression, relapse, primary graft failure, death from any cause, or last follow-up. The comparisons of GvHD between groups were assessed *via* cumulative incidence estimates and Cox proportional hazards using Fine and Gray’s method. The univariate and multivariate analyses were performed to identify factors associated with GvHD and GRFS. Factors with *p* < 0.1 in univariate analysis were included in multivariate analysis. A multivariate analysis was performed with a Cox proportional hazards regression model. For GRFS, the Wald test of significance was used. For cGvHD and moderate to severe cGvHD, as logLik converged before TNF fold increased and the coefficient became <3.9 × 10^–10^ in a Cox model, the Wald test of significance became unreliable. Thus, a likelihood ratio test was used to assess whether a model consisting of TNF fit better. A receiver operating characteristic (ROC) analysis was performed to evaluate the best threshold in the terms of specificity and sensitivity. Statistical analyses, such as parametric tests, non-parametric tests, and chi-square analysis, were performed using GraphPad Prism (GraphPad Software, San Diego, CA, United States). A statistical analysis of survival and the cumulative incidence of events were performed using R 4.0.4 software downloaded from https://www.r-project.org/.

## Results

### Patient Characteristics

The characteristics of 37 patients are summarized in Table 1. With a median follow-up duration of 308 days (range 41–562 days), the estimated 1-year OS for the whole cohort was 85.8%, and the estimated GRFS was 37.6%. The 1-year NRM of the whole cohort was 10.8%, and the cumulative incidence of relapse (CIR) was 13.5%. The incidence of grade II–IV aGvHD was 13.5%, the incidence of cGvHD was 29.7%, and that of moderate to severe cGvHD was 16.2% (Table 2).

**TABLE 1:** Patient characteristics.

	*N*	%
All patients	37	
Male	26	70.3
Female	11	29.7
Age in years	37 (range 18–59)	
**Disease**		
AML	19	51.4
MDS	5	13.5
ALL	7	18.9
Lymphoma	4	10.8
GS	1	2.7
Mixed lineage AL	1	2.7
**Disease status**		
CR	25	67.6
PR	2	5.4
Not in remission	7	18.9
Untreated	3	8.1
**Disease risk index**		
Low risk MRD+	1	2.7
Intermediate risk	23	62.2
High risk/very high risk	11	29.7
Not evaluable in DRI	2	5.4

*AML, acute myeloid leukemia; MDS, myelodysplasia; ALL, acute lymphoblastic leukemia; GS, granulocytic sarcoma; AL, acute leukemia; CR, complete remission; PR, partial remission; MRD, measurable residual disease; DRI, disease risk index.*

**TABLE 2:** The summary of transplants and clinical outcomes.

	*N*	%
Total transplantations	37	
**Conditioning regimen**		
FLU BU MEL based	19	51.4
FLU BU based	12	32.4
TBI FLU VP16	1	2.7
Sequential conditioning	5	13.5
**Donor type**		
Child	15	40.5
Parent	15	40.5
Mismatched sibling	6	16.2
Collateral related donors	1	2.7
**Median infused cell number**		
CD34 (×10^6^/kg)	7.2 (3.9–11.4)	
MNC (×10^8^/kg)	7.1 (2.5–12.2)	
CD3+ cells (×10^8^/kg)	5.1 (1.9–8.4)	
**Transplantation outcome**		
1-year NRM	10.8% (5.6–16%)	
1-year CIR	13.5% (7.3–19.7%)	
1-year OS	85.8% (74.9–98.3%)	
1-year GRFS	37.6% (23.2–60.9%)	
**GvHD**		
Grade II-IV aGvHD	13.5% (7.8–19.2%)	
cGvHD	29.7% (22.0–37.4%)	
Moderate to severe cGvHD	16.2% (10.0–22.4%)	

*FLU, fludarabine; BU, busulfan; MEL, melphalan; TBI, total body irradiation; VP16, etoposide; MNC, mononuclear cell; NRM, non-relapse mortality; CIR, cumulative incidence of relapse; OS, overall survival; GRFS, graft versus host disease and relapse-free survival; GvHD, graft versus host disease.*

### Haplo-Fever After Graft Infusion

In total, 33 patients (89.2%) developed febrile neutropenia between day 0 and day +3 (4/33 at day 0, 19/33 on day +1, 8/33 on day +2, and 2/33 on day +3). Most of these patients became afebrile immediately after the use of PTCy (28/33 on day +5 and 2/33 on day +6), with only 3 patients developing persistent fever after day +7. The median duration of fever was 4 days (range 1–6 days). The median peak temperature was 39.5°C (range 38.0–40.7°C). Peak fever was significantly higher in patients with donors with 5/10 HLA allele mismatches than in patients with donors with ≤4 mismatches (*p* = 0.011). In contrast, neither the number of CD3^+^ cells nor the number of mononuclear cells infused were associated with peak haplo-fever (Supplementary Figure 1). Only one patient who underwent a sequential conditioning regimen had fever that started before graft infusion and persisted during the first few days post-graft infusion. This same patient had the microbiological documentation of bloodstream infection on day +2. There was no microbiological documented infection in any other patient. Only four patients remained afebrile between day 0 and day +6, among whom two patients were 9/10 loci matched with their donor.

**FIGURE 1:**
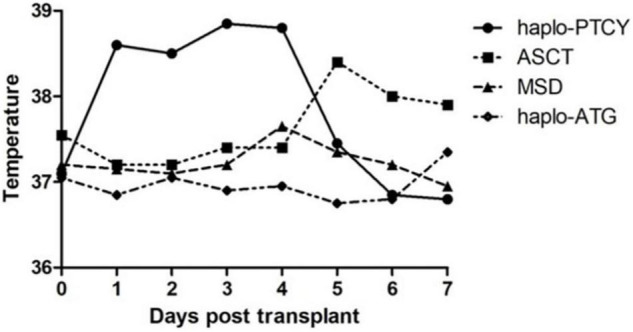
The temperature profile of patients undergoing hematopoietic stem cell transplantation (HSCT) of various types from June 2019 to December 2020 [haplo-post-transplantation cyclophosphamide (haplo-PTCY) *n* = 37; macthed sibling donor (MSD) *n* = 20; autologous stem cell transplant (ASCT) *n* = 18; haplo-anti-thymoglobulin *n* = 4].

This unique pattern of early fever after graft infusion was not observed in patients undergoing autologous transplantation, allo-HSCT from HLA-matched donors, or haplo-HSCT with pre-transplant anti-thymoglobulin as GvHD prophylaxis during the same period at our center (Figure 1). Based on this observation, we defined early fever after the infusion of haplo-identical graft and before PTCy as “haplo-fever.”

### Correlation Between Haplo-Fever and Transplantation Outcomes

The associations between haplo-fever and transplantation outcomes were evaluated. Of the 4 patients who did not develop haplo-fever, 2 failed to obtain either neutrophil engraftment or autologous hematologic recovery by day +28, whereas no primary graft failure was documented in patients with haplo-fever (*p* = 0.009).

The patients were dichotomized into high-fever and low-fever groups based on the median peak temperature (39.5°C). Neither high nor low fever was significantly associated with the risk of aGvHD (*p* = 1), but high-fever patients tended to have lower cGvHD (*p* = 0.07), and low-fever patients tended to have moderate to severe cGvHD (*p* = 0.08). CIR, NRM, and OS were comparable in both groups, but patients in the low-fever group had significantly inferior GRFS (*p* = 0.04, Supplementary Figure 2).

### Cytokine Profile During Haplo-Fever

Cytokine profiles from day 0 to day +7 were analyzed in detail. The levels of multiple cytokines started to increase on day +1, peaked between day +2 and day +4, and returned to baseline on day +5 or +6 (right after PTCy). Among these cytokines, IFNγ, IL6, and IL2R increased most significantly during haplo-fever, with respective peak fold increases (cytokine level at peak day/baseline level on day 0) of 40.7, 9.6, and 5.5. The levels of IL1β, IFNα, IL4, and IL12p70 did not change substantially during the haplo-fever.

Cytokine levels in patients with no haplo-fever, low haplo-fever, and high haplo-fever were compared. Since cytokine levels did not differ significantly in patients with no haplo-fever and low haplo-fever, these patients were combined and then the combined group was compared with patients with high haplo-fever. We focused on fold increase on day +4 because most cytokines reached a peak on that day. IL2R (*p* = 0.004), IL6 (*p* = 0.021), IL10 (*p* = 0.029), IFNγ (*p* = 0.018), IL2 (*p* = 0.018), and IL17 (*p* = 0.003) were significantly higher in patients with high haplo-fever (Supplementary Figure 3).

### Correlations Between Fold Increases in Cytokines and Transplant Outcomes

**FIGURE 2:**
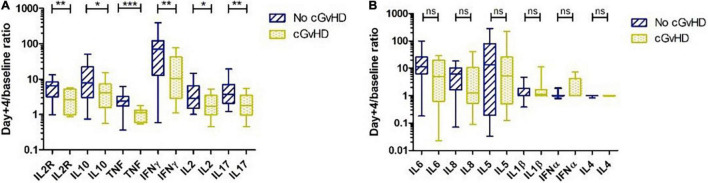
Cytokine profile and chronic graft versus host disease (cGvHD). **(A)** Patients without cGvHD had significantly higher levels of interleukin (IL) 2R, IL10, tumor necrosis factor (TNF), IFNγ, IL2, and IL17 on day +4 than patients with cGvHD. **(B)** IL6, IL8, IL5, IL1β, interferon (IFN) α, and IL4 on day +4 were comparable in patients with and without cGvHD. *p < 0.05, **p < 0.01, ***p < 0.001, ns, not significant.

To investigate whether cytokine levels were associated with the occurrence of GvHD, fold increases in multiple cytokines were evaluated on day +4. There were no significant differences in patients with and without aGvHD (data not shown). In patients without cGvHD, the fold increases in IL2R (*p* = 0.004), IL10 (*p* = 0.017), TNF (*p* < 0.0001), IFNγ (*p* = 0.007), IL2 (*p* = 0.023), and IL17 (*p* = 0.004) on day +4 were significantly higher than those in patients with documented cGvHD (Figure 2A). Fold increases in all other cytokines, such as IL6, IL8, IL5, IL1β, IFNα, and IL4, on day +4 were comparable in patients with and without cGvHD (Figure 2B).

**FIGURE 3:**
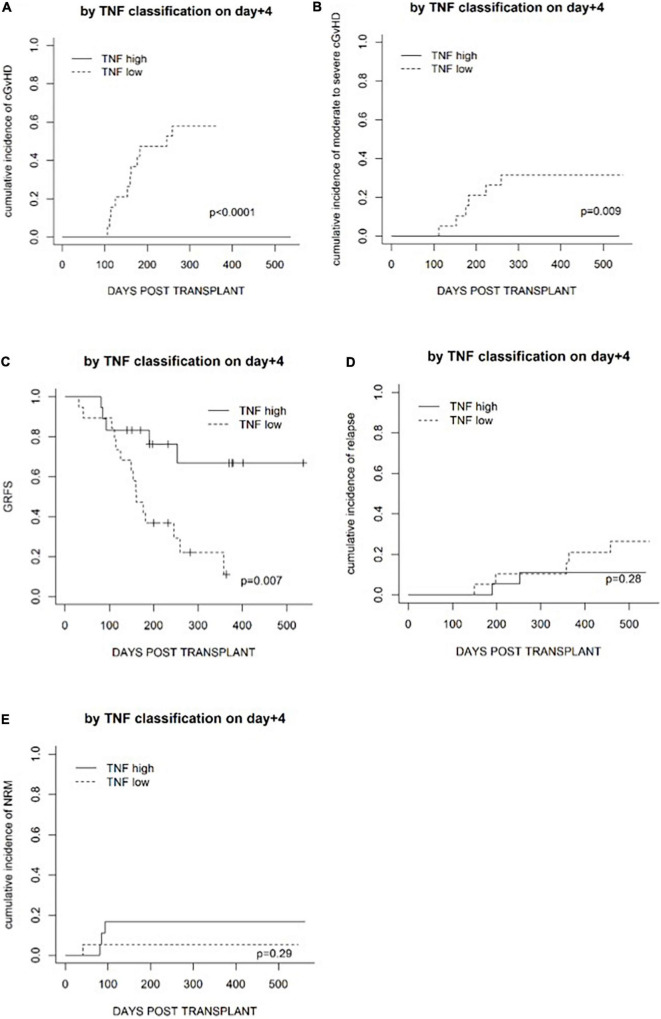
Transplantation outcomes in patients in a TNF-high group and a TNF-low group based on the median level on day +4. **(A)** The cumulative incidence of cGvHD. **(B)** The cumulative incidence of moderate to severe cGvHD. **(C)** GvHD and relapse-free survival (GRFS). **(D)** The cumulative incidence of relapse. **(E)** The cumulative incidence of non-relapse mortality.

Based on the above-described data, patients were further classified into cytokine-high or cytokine-low groups according to the median fold increase in cytokines on day +4, to investigate whether cytokine fold increase could predict the risk of GvHD. No cytokine profile predicted the risk of aGvHD, but high IL2R and TNF fold increase on day +4 were associated with a lower risk of cGvHD and a lower risk of moderate to severe cGvHD (Table 3). Higher TNF was associated with a lower cumulative incidence of moderate to severe cGvHD (*p* = 0.009) (Figure 3).

**TABLE 3:** The predictors of cGvHD.

Cytokine	cGvHD incidence	RR	95% CI	*p*
IL2R high vs. low on day +4	6.3 vs. 40.0%	0.44	0.24–0.80	0.037
TNF high vs. low on day +4	0 vs. 61.1%	0.28	0.15–0.53	<0.0001

**Cytokine**	**Moderate to severe cGvHD incidence**	**RR**	**95% CI**	***p***

IL2R high vs. low on day +4	0 vs. 26.7%	0.41	0.26–0.64	0.04
TNF high vs. low on day +4	0 vs. 31.6%	0.42	0.28–0.63	0.02

*IL2R, interleukin 2 receptor; TNF, tumor necrosis factor; cGvHD, chronic graft versus host disease; RR, relative risk; CI, confidence interval.*

Whether fold increases in cytokines on day +4 could predict transplantation outcomes in the terms of OS, relapse (CIR), NRM, and GRFS was investigated. Fold increases in IL2R and TNF on day +4 were not associated with CIR or NRM (Figure 3). High TNF fold increase on day +4 was associated with superior GRFS (*p* = 0.007) but not OS (*p* = 0.3), and fold increase in IL2R on day +4 was not associated with GRFS (*p* = 0.4) or OS (*p* = 0.4).

### Tumor Necrosis Factor Fold Increase as a Predictor of Transplantation Outcome: Receiver Operating Characteristic Analysis, Univariate Analysis, and Multivariate Analysis

**FIGURE 4:**
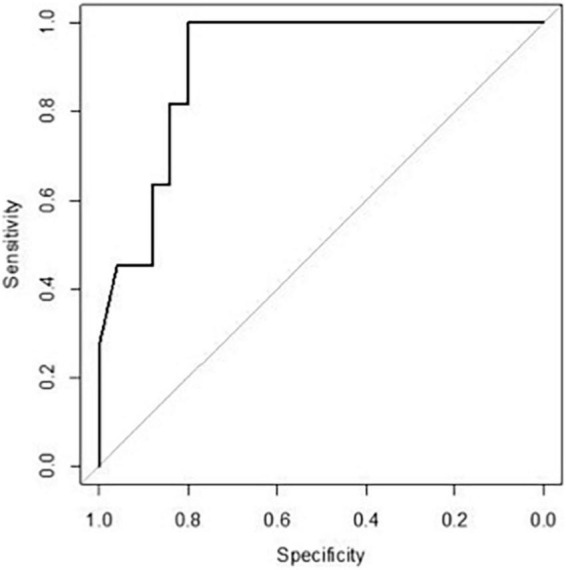
A receiver operating characteristic (ROC) curve of TNF fold increase on day +4.

**FIGURE 5:**
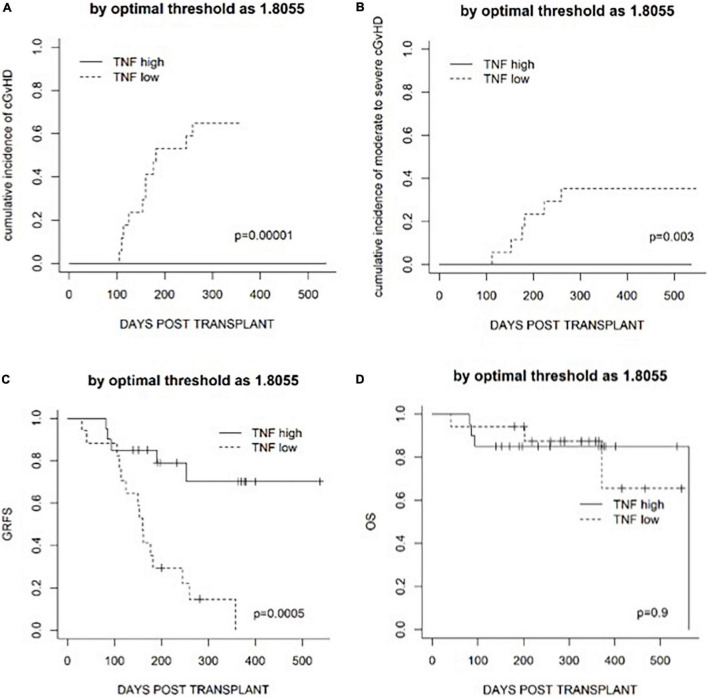
Transplantation outcomes in patients classified according to the optimal threshold defined by an ROC curve analysis. **(A)** The cumulative incidence of cGvHD. **(B)** The cumulative incidence of moderate to severe cGvHD. **(C)** GRFS. **(D)** Overall survival (OS).

Since fold increase in TNF on day +4 was correlated with the risk of cGvHD, an ROC curve analysis was performed. The area under the curve of TNF fold increase on day +4 was 0.909 [95% confidence interval (*CI*) 0.82–1.00] (Figure 4). The fold increase at 1.8055 was the best threshold to predict the risk of cGvHD and GRFS (as shown in Figure 5).

Based on the heterogeneity of patients enrolled in the terms of diagnosis, disease status at transplantation, and the intensity of conditioning regimen, which may affect the risk of GvHD (16–18), univariate and multivariate analyses were performed. In the univariate analysis, less mismatched HLA loci were associated with superior GRFS, whereas active disease at the time of transplantation and an intensified conditioning regimen was associated with the higher cumulative incidence of moderate to severe cGvHD (Table 4). Active disease (*p* = 0.017) and the use of an intensified conditioning regimen (*p* = 0.015) were also associated with a lower fold increase in TNF on day +4 (Supplementary Figure 4). Factors with *p* < 0.1 in the univariate analysis were included in the multivariate analysis to further investigate the predictive value of the fold increase in TNF. Since all the patients who underwent chronic GvHD were in the TNF low group, logLik converged before factor ‘TNF fold increase,’ making the coefficient <10^–8^ in a Cox model both for cGvHD and for moderate to severe cGvHD. Thus, the Wald test of significance became unreliable. To further assess whether a model consisting of TNF fits better, we used the likelihood test in this case. Overall, a fold increase in TNF lower than an ROC-based threshold of 1.8055 was an independent predictive factor for the higher cumulative incidence of cGvHD, moderate to severe cGvHD, and worse GRFS in the multivariate analysis (Table 4).

**TABLE 4:** The univariate and multivariate analysis of transplant outcomes.

	Univariate analysis	Multivariate analysis		
*GRFS*	*p*	HR	95% CI of HR	*P*
TNF fold increase lower than 1.8055	0.0005***	3.73	2.98–4.48	0.02*
Less than 5 loci mismatched	0.01*	0.93	0.18–1.68	0.10
Active disease at transplant	0.1	NA	NA	NA
Intensified conditioning regimen	0.3	NA	NA	NA
Low haplo-fever peak	0.02*	1.63	0.92–2.34	0.33
*cGvHD*	*p*	HR	95% CI of HR	*P*
aGvHD	0.14	NA	NA	NA
TNF fold increase lower than 1.8055	0.00001***	4.30 × 10^–10^	1.42 × 10^–10^–1.30 × 10^–9^	0.0003***†
Less than 5 loci mismatched	0.07	1.44	0.36–5.84	0.61
Active disease at transplant	0.11	NA	NA	NA
Intensified conditioning regimen	0.09	0.99	0.29–3.32	0.98
Low haplo-fever peak	0.09	0.80	0.20–3.2	0.75
*Moderate to severe cGvHD*	*p*	HR	95% CI of HR	*P*
aGvHD	0.59	NA	NA	NA
TNF fold increase lower than 1.8055	0.003**	1.09 × 10^–9^	1.83 × 10^–10^–6.51 × 10^–9^	0.02*†
Less than 5 loci mismatched	0.18	NA	NA	NA
Active disease at transplant	0.005**	3.54	0.71–17.79	0.12
Intensified conditioning regimen	0.03*	1.48	0.31–7.2	0.62
Low haplo-fever peak	0.09	6.48	0.05–8.86	0.75

*HR, hazard ratio; GRFS, graft versus host disease and relapse free survival; cGvHD, chronic graft versus host disease; CI, confidence interval; NA, not analyzed.*

^†^LogLik converged before TNF fold increase, the coefficient goes to <10^–8^ in a Cox model both for cGvHD and for moderate to severe cGvHD, the Wald test of significance became unreliable. Instead, we performed a likelihood ratio test and a model consisting of TNF fit significantly better. The p-value shown here represents the comparison of the model with and without TNF. The HR and 95% CI of HR represents the results of a Wald test, as in the case of other factors. *p < 0.05, **p < 0.01, ***p < 0.001. Bold values are statistically significant results with the likelihood ratio test.

## Discussion

The current study investigated cytokine profiles during haplo-fever, and their clinical significance. Multiple cytokines shared the same upward and downward pattern in conjunction with haplo-fever, indicating that haplo-fever is a CRS associated with the activation of infused donor T cells, which were eliminated by subsequent PTCy treatment. The extent of elevation of the cytokines, such as TNF, could be used as an independent predictor of transplantation outcome in the terms of cGvHD, moderate to severe cGvHD, and GRFS.

Early fever after allogeneic graft infusion was frequently observed in patients with haplo-identical donors who were administered PTCy as GvHD prophylaxis, but it rarely occurred in other types of transplantation. To further investigate this issue, we compared cytokine levels early after graft infusion in patients with different transplant settings, such as auto-HSCT, allo-HSCT from an HLA-matched donor, and haplo-HSCT with or without PTCy. Though the number of patients was limited, cytokines in the haplo-PTCy setting were generally more significantly increased than they were in all other transplant settings (Supplementary Figure 5). This observation suggested that the cytokine release during haplo-fever was unique and not simply due to toxicity associated with the conditioning regimen, such as endothelial injury. Infection was the major cause of febrile events in allo-HSCT, but microbiological evidence was documented in very few cases during haplo-fever. More importantly, most patients became afebrile right after the PTCy, suggesting a more likely immune-based mechanism. Based on the cytokine profile data, we speculated that haplo-fever could be a CRS reflecting an early allo-reactivation of infused repleted donor T cells against the haplo-identical recipient because no immunosuppressive agent was given before the infusion of haplo-identical graft in the PTCy setting. This may explain the following observations in the study: (1) the severity of haplo-fever or allo-reactivation was correlated with the extent of donor-recipient HLA mismatch; and (2) haplo-fever or allo-reactivation recovered right after PTCy treatment.

There are several reports of early fever in haplo-HSCT as part of CRS. Abboud et al. (10) reported an incidence of the CRS of 87% within 14 days after haplo-HSCT. The CRS was mostly mild (grade 1 or 2) with very few grade 3 cases requiring immunosuppressive treatment. In that study, however, patients with CRS developed grade II–IV acute GVHD more frequently (60 vs. 28.6%, *p* = 0.012) and severe but not mild CRS was associated with increased NRM and inferior OS. Imus et al. (19) reported that the occurrence of severe CRS in the setting of haplo-HSCT using PTCy was associated with delayed hematological recovery, a higher incidence of NRM, and poorer OS. In addition, they reported an association between severe CRS and low risk of moderate to severe cGvHD.

There were several differences between the present study and previous studies. First, there was no severe CRS documented in our series. Older age and the use of PBSC as the graft source have been considered risk factors for severe CRS (19, 24), and the relatively younger age (median 37 years) of our study cohort may have spared the patients from severe CRS. Since bone marrow was not regularly used at our center, the impact of graft source on the occurrence and severity of haplo-fever was not evaluated in the study. Second, there were differences in the definition and timing of early fever vs. CRS. In the current study, haplo-fever was defined as fever early after PBSC infusion, specifically from day 0 to day +6, because fever together with most cytokines decreased to baseline level right after PTCy. Notably, however, other studies have included patients with CRS-associated symptoms until the peri-engraftment period (10, 13), in which case patients with engraftment syndrome or early-onset aGvHD may not be able to be ruled out. Third, cytokine elevation was not associated with aGvHD in the present study. aGvHD is generally thought to be evoked by the activation of immune cells secondary to tissue damage caused by conditioning and infection (20), whereas the recipient’s microbiota and its metabolites also play critical roles in mediating GvHD (21). The observed simultaneous decrease in cytokines and temperature during haplo-fever right after PTCy suggest that CRS associated with haplo-fever may be an independent event that is unlikely to contribute to aGvHD. Significant cytokine elevation during haplo-fever was strongly associated with a low incidence of cGvHD and with moderate to severe cGvHD. This was concordant with the hypothesis that the early cytokine release cascade reflected the activation of infused donor allo-reactive T cells, which were selectively eliminated by PTCy, leading to a lower risk of cGvHD (22, 23).

To the best of our knowledge, this is the first study focusing on cytokine profiles during early fever in haplo-HSCT with PTCy and their clinical significance. TNF may be a predictor of both cGvHD and GRFS based on an ROC curve analysis. With respect to non-specific inflammatory biomarkers, C-reactive protein was linearly associated with TNF fold increase (Supplementary Figure 6) but not with the incidence of cGvHD, whereas procalcitonin was not correlated with TNF fold increase or transplant outcomes.

The present study had inevitable limitations due to its retrospective nature, the limited number of patients, and the relatively short follow-up period. An external group of patients is required to confirm our results. Cytokines reportedly related to the risk of GvHD, such as IL33 and IL15, were not included in our cytokine panel. Nevertheless, the study was the first to investigate cytokine profiles and their clinical significance during haplo-fever in haplo-HSCT with PTCy. The data suggested an association between early haplo-fever and cytokine release, and that TNF may serve as a surrogate predictor of transplantation outcomes in the terms of cGvHD and GRFS. Further research with a larger independent cohort of patients is warranted.

## Data Availability Statement

The raw data supporting the conclusions of this article will be made available by the authors, without undue reservation.

## Ethics Statement

The studies involving human participants were reviewed and approved by the Human Ethics Committee of Ruijin Hospital. The patients/participants provided their written informed consent to participate in this study. Written informed consent was obtained from the individual(s) for the publication of any potentially identifiable images or data included in this article.

## Author Contributions

LiniW, BD, WG, and JW performed the transplants in patients in the prospective clinical trials, collected the patient data, and wrote the manuscript. LiniW, RW, and MW performed the statistical analysis. LingW and JJ supervised the prospective clinical trials and followed-up with the patients. DB and JH designed and supervised the study, and wrote the manuscript. All authors contributed to the article and approved the submitted version.

## Conflict of Interest

The authors declare that the research was conducted in the absence of any commercial or financial relationships that could be construed as a potential conflict of interest.

## Publisher’s Note

All claims expressed in this article are solely those of the authors and do not necessarily represent those of their affiliated organizations, or those of the publisher, the editors and the reviewers. Any product that may be evaluated in this article, or claim that may be made by its manufacturer, is not guaranteed or endorsed by the publisher.
