# Supplementary material for: Clinical Significance of Haplo-Fever and Cytokine Profiling After Graft Infusion in Allogeneic Stem Cell Transplantation From Haplo-Identical Donors
Source: Front Med (Lausanne). 2022 Apr 7;9:820591. doi: 10.3389/fmed.2022.820591 (PMC9021571; doi:10.3389/fmed.2022.820591)
Supplement: Supplementary file 1 [file Data_Sheet_1.docx]

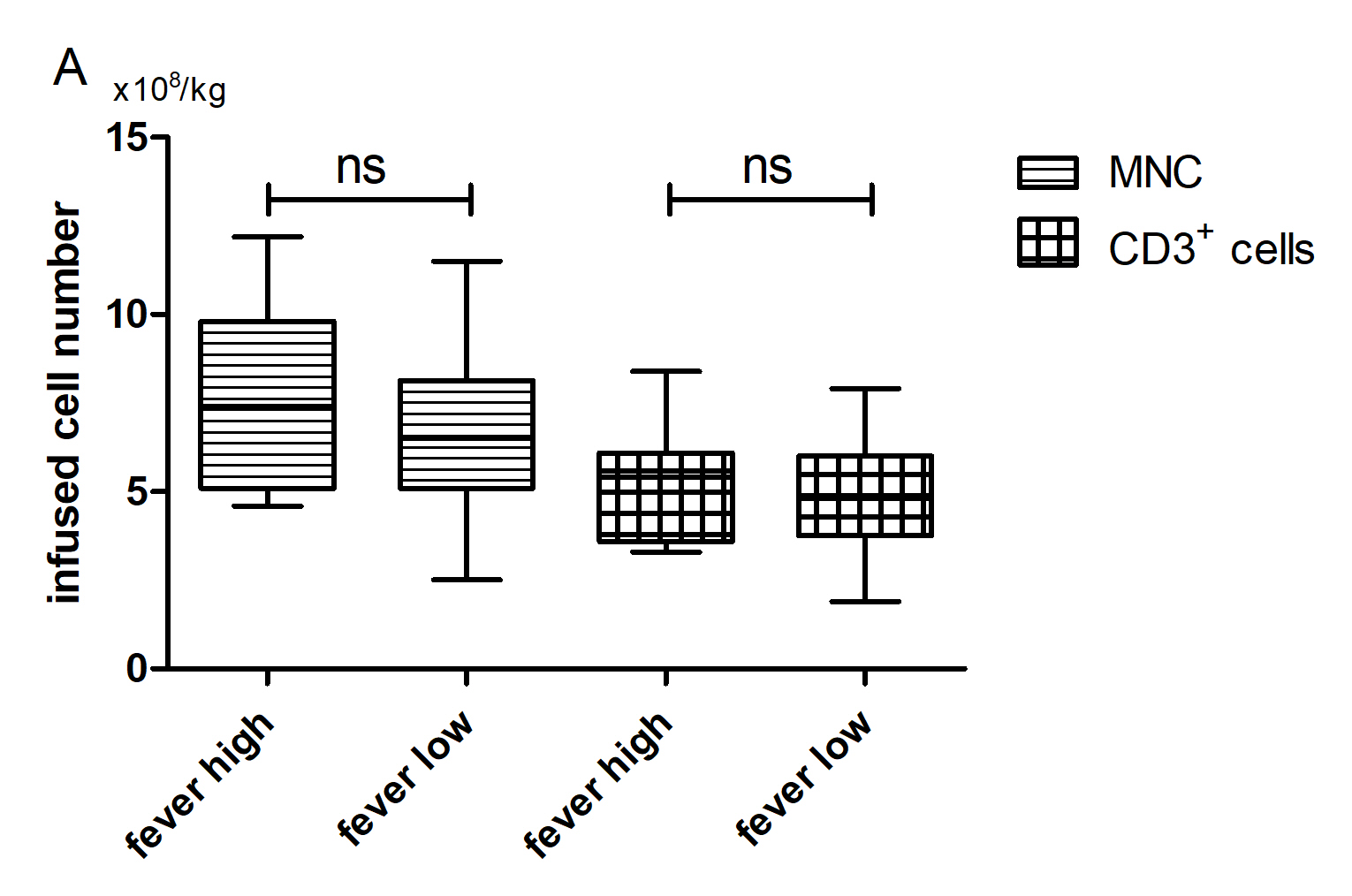

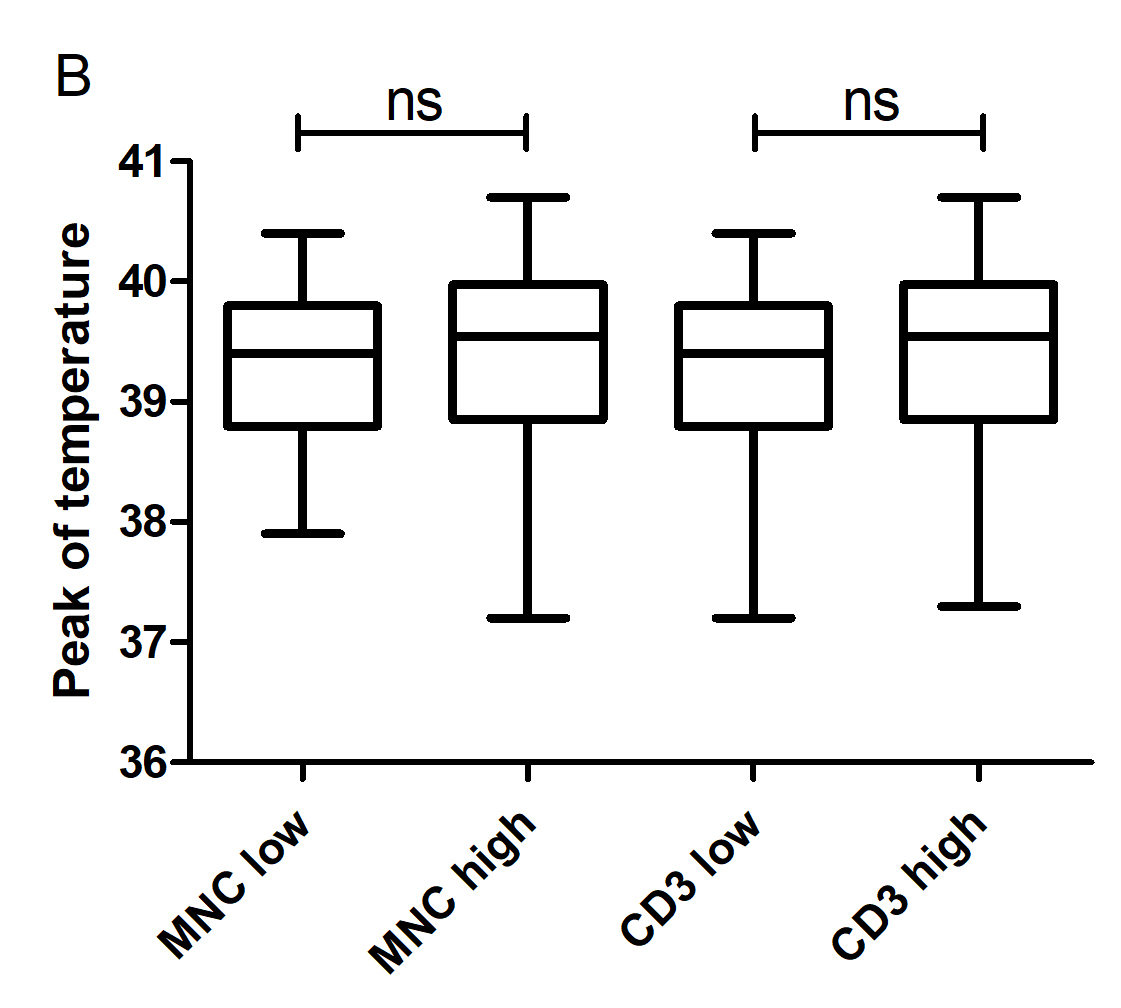


**Supplementary Figure 1**. Numbers of cells infused and the peak of haplo-fever. （A）comparison of infused cell number according to peak of haplo-fever. (B) comparison of peak of haplo-fever according to infused cell number.















**Supplementary Figure 2.** Peak of haplo-fever and outcomes of transplantation. Incidence of cGvHD, NRM, CIR and GRFS of patients from high fever group and low fever group.


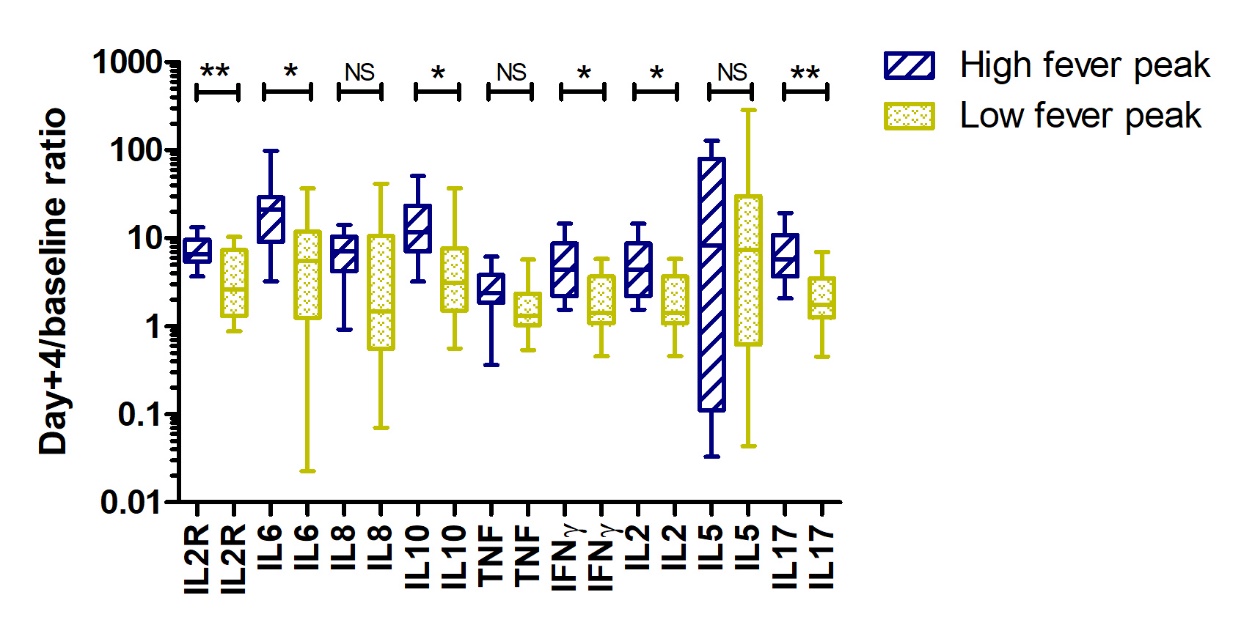


**Supplementary Figure 3.** Peak of haplo-fever and cytokine profiles. Patients with higher peak of haplo-fever had greater elevation of IL2R, IL6, IL10, IFNγ, IL2 and IL17 on day +4. *: p<0.05, **: p<0.01, ***: p<0.001


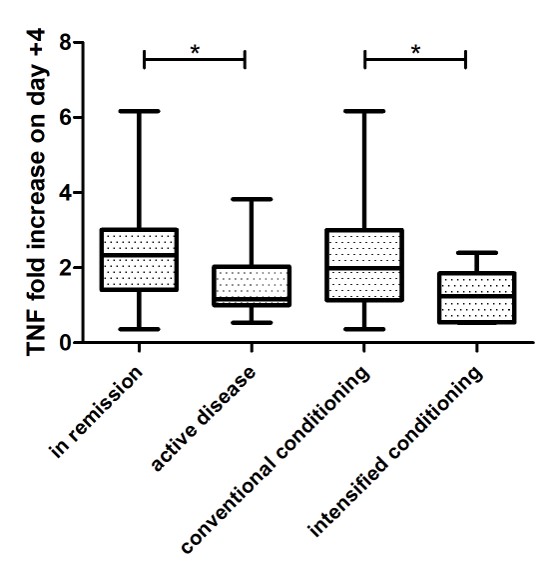


**Supplementary Figure 4.** Correlation of disease status and conditioning intensify with TNF fold increase.


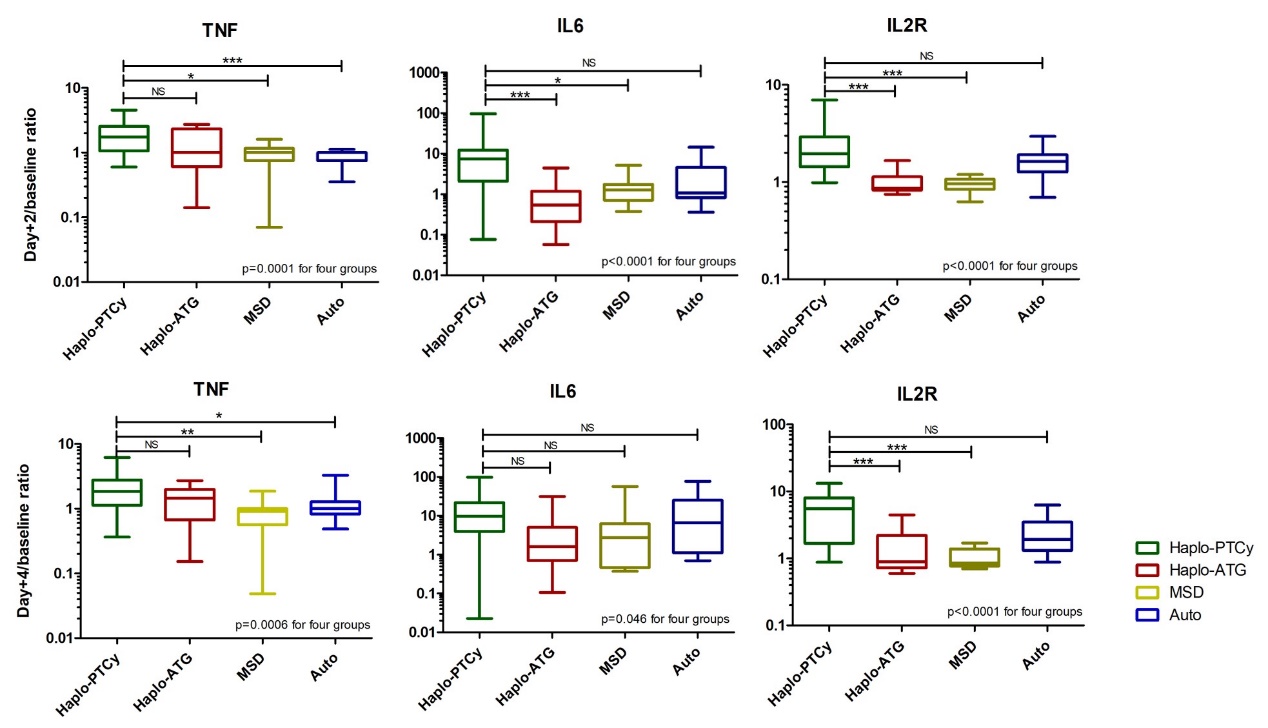


**Supplementary Figure 5.** Levels of TNF, IL6 and IL2R on day+2 and day+4 among patients undergoing different transplant settings (Haplo-PTCy N=37, Haplo-ATG N=4, MSD N=9, Auto N=13).


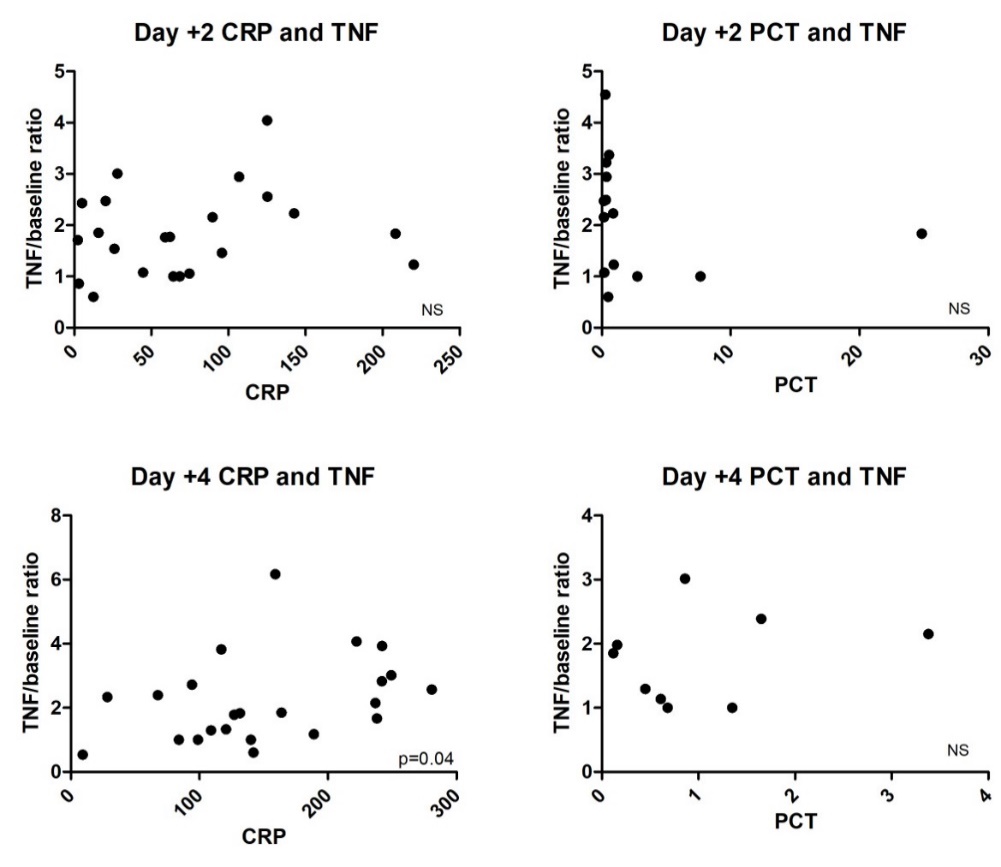


**Supplementary Figure 6.** Correlation between C-reactive protein, procalcitonin and TNF on day+2 and day+4.
